# Anti-SARS-CoV-2 potential of *Cissampelos pareira* L. identified by connectivity map-based analysis and in vitro studies

**DOI:** 10.1186/s12906-022-03584-3

**Published:** 2022-04-22

**Authors:** Madiha Haider, Vivek Anand, M. Ghalib Enayathullah, Yash Parekh, Sushma Ram, Surekha Kumari, Anmol, Gayatri Panda, Manjari Shukla, Dhwani Dholakia, Arjun Ray, Sudipta Bhattacharyya, Upendra Sharma, Kiran Kumar Bokara, Bhavana Prasher, Mitali Mukerji

**Affiliations:** 1 Genomics & molecular medicine, CSIR-Institute of Genomics and Integrative Biology, Delhi, 110007 India; 2 Academy of Scientific and Innovative Research, Ghaziabad, Uttar Pradesh 201002 India; 3 CSIR-Center for Cellular and Molecular Biology, Hyderabad, Telangana 500007 India; 4 Chemical Technology Division, CSIR-Institute of Himalayan Bioresource Technology, Palampur, Himachal Pradesh 176 061 India; 5 Department of Computational Biology, Indraprastha Institute of Information Technology, Delhi, India; 6 Department of Bioscience & Bioengineering, Indian Institute of Technology Jodhpur, NH 62, Karwar, Rajasthan 342037 India; 7 Centre of Excellence for Applied Developments of Ayurveda Prakriti and Genomics, CSIR’s Ayurgenomics Unit TRISUTRA, CSIR-IGIB, New Delhi, 110025 India

**Keywords:** *Cissampelos pareira* L., SARS-CoV-2, COVID-19, Antivirus, Whole plant extract, Connectivity map, BALF

## Abstract

**Background:**

Viral infections have a history of abrupt and severe eruptions through the years in the form of pandemics. And yet, definitive therapies or preventive measures are not present. Herbal medicines have been a source of various antiviral compounds such as Oseltamivir, extracted using shikimic acid from star anise (*Illicium verum*) and Acyclovir from *Carissa edulis* are FDA (Food and Drug Administration) approved antiviral drugs. In this study, we dissect the anti-coronavirus infection activity of *Cissampelos pareira* L (*Cipa*) extract using an integrative approach.

**Methods:**

We analysed the signature similarities between predicted antiviral agents and *Cipa* using the connectivity map (https://clue.io/). Next, we tested the anti-SARS-COV-2 activity of *Cipa *in vitro. Molecular docking analyses of constituents of with key targets of SARS-CoV2 protein viz. spike protein, RNA‑dependent RNA‑polymerase (RdRp) and 3C‑like proteinase. was also performed. A three-way comparative analysis of *Cipa* transcriptome, COVID-19 BALF transcriptome and CMAP signatures of small compounds was also performed.

**Results:**

Several predicted antivirals showed a high positive connectivity score with *Cipa* such as apcidin, emetine, homoharringtonine etc. We also observed 98% inhibition of SARS-COV-2 replication in infected Vero cell cultures with the whole extract. Some of its prominent pure constituents e.g. pareirarine, cissamine, magnoflorine exhibited 40–80% inhibition. Comparison of genes between BALF and *Cipa* showed an enrichment of biological processes like transcription regulation and response to lipids, to be downregulated in *Cipa* while being upregulated in COVID-19. CMAP also showed that Triciribine, torin-1 and VU-0365114–2 had positive connectivity with BALF 1 and 2, and negative connectivity with *Cipa*. Amongst all the tested compounds, Magnoflorine and Salutaridine exhibited the most potent and consistent strong in silico binding profiles with SARS-CoV2 therapeutic targets.

**Supplementary Information:**

The online version contains supplementary material available at 10.1186/s12906-022-03584-3.

## Introduction

SARS-CoV-2, the severe acquired respiratory syndrome agent coronavirus 2, has taken many lives in the past year and is continuing to create an unsafe environment. Along with numerous mutations emphasizing the quasispecies nature of the virus [1, 2], fast transmission and a wide range of symptoms, lack of a definite therapeutic intervention has made this virus all the more deadly. Due to the ongoing mutations new variants such as Delta variants emerge with higher or different COVID-19 manifestations [3]. Many studies have been conducted in order to recognize small compound therapeutics effective against SARS-CoV-2. Interestingly, some estrogen receptor modulators and protein synthesis inhibitors having potential antiviral against SARS-CoV2 have also been identified [4]. It has been shown that *ESR1* as a drug target can modulate certain coronavirus associated genes. A group recently demonstrated the downregulation of *ACE2* (Angiotensin-converting enzyme 2) by estrogen [5]. It has been shown that ACE-2 protein on the epithelial surface is the primary entry receptor for SARS-CoV-2 [6].

Several herbal medicines and natural sources have been used to isolate antiviral compounds. Some of the examples of FDA approved antivirals from natural sources include Oseltamivir from star anise (Illicium verum) and Acyclovir from Carissa eduli [7]. *Cissampelos pareira* L. is a commonly used hormone modulator which is used to treat reproductive disorders and fever in Ayurveda and also other healing systems. It has also been found to inhibit three serotypes of dengue [8] and its effect on various hormones has also been evidenced [9]. In a previous study we have observed that *Cipa* can act as both, a protein synthesis inhibitor and an estrogen receptor inhibitor [10]. Many of the drugs positively connected with *Cipa* have been reported to be a potential antiviral agent. Since there were several overlaps between the therapeutics predicted to be effective against SARS-CoV-2 and our formulation, we decided explore the repurposing potential of *Cipa* for this current pandemic. We found that Cipa can inhibit viral replication and Cipa compounds could directly to the SARS-CoV-2 spike protein, RNA‑dependent RNA‑polymerase (RdRp) and 3C‑like proteinase.

## Material and methods

### Transcriptome meta-analysis

We obtained the raw RNA sequencing data from SARS-CoV-2 patients Broncho-alveolar lavage fluid (BALF) 1 and 2 from the recent publications and analyzed them inhouse (Supplementary information 1.1). The gene expression data for *Cissampelos pareira* L. was taken from [10], which can be accessed at GSE156445.

### Functional enrichment and connectivity map of the differentially expressed genes

The differentially expressed genes were analyzed for functional enrichment using enrichr [11] and for similar signatures using clue.io [12]. The results were then compared with *Cipa* to find intersections between gene ontologies, enriched gene sets, and connectivity map perturbations between upregulated genes of BALF 1 and 2 and downregulated genes of *Cipa* and between downregulated genes of BALF 1 and 2 and upregulated genes of *Cipa*. Enrichments were also done for the genes whose knockdown signatures score > 90 connectivity with *Cipa* and then compared with the upregulated processes and gene sets in SARS-CoV-2.

### Collection of plant material and preparation of extract for in vitro inhibition assays

Whole plant of *Cissampelos pareira* L was collected from the campus of CSIR-IHBT, Palampur, HP, India (alt. 1350 m) following biodiversity guidelines. The identification of the plant material was done by a taxonomy expert in CSIR-IHBT, Palampur and a voucher specimen (no. PLP16688) was deposited in the herbarium of CSIR-IHBT, Palampur, HP-176,061, India. The plant has been obtained and isolated as per CSIR, India guidelines (details for extract preparation in Supplementary information 1.2). The pure molecules from roots of the plant were isolated as reported recently [13].

### Cell culture, viral infection and drug treatment for inhibition of SARS-COV-2 by *Cipa*

The effect of PE50 and PER was tested against the SARS-CoV2 (ASTM, 2015) in a 96-well tissue culture plates that was seeded with Vero Cells which were obtained from ATCC (CCL-81) 24 h prior to infection with SARS-CoV2 (Indian/a3i clade/2020 isolate) in BSL3 facility. After treatment, RNA was isolated using MagMAXTM Viral/Pathogen Extraction Kit (Applied Biosystems, Thermofisher) according to the manufacturer’s instructions. The details of the experiment and RNA isolation protocol are given in the Supplementary information 1.3.

### TaqMan real-time RT-PCR assay for detection of SARS-CoV-2

**T**he detection of genes specific to SARS-CoV2 was done using COVID-19 RT-qPCR Detection Kit (Fosun 2019-nCoV qPCR, Shanghai Fosun Long March Medical Science Co. Ltd.) according to the manufacturer’s instructions (details in Supplementary information 1.4). The calculations for the relative viral RNA content and log reduced viral particles was calculated using the linear regression equation obtained using the RNA extracted from the known viral particles by RT-qPCR, using N, E and ORF1ab genes specific to SARS CoV2 virus. from the test sample [14].

### Docking of *Cipa* constituent compounds to key SARS-CoV-2 drug targets

The catalytic groove of SARS-CoV2 3C-like protease (3CL-pro) was identified from the high resolution crystal structure of the enzyme (PDB Id: 6WTJ) [15]. Autodock Vina [16] which uses Lamarckian Genetic Algorithm method was used to perform in silico docking simulations of the Cipa bioactive compounds at the active site of 3CL-pro. Molecular Graphic Laboratory tools (MGL tools) were used to add polar hydrogens, Gasteiger charges and finally to prepare the pdbqt file of the receptor protomer. The coordinate files of the Cipa bioactive compounds were retrieved from PubChem [17] in sdf format and was later converted to pdb format using Pymol [18]. Again, the MGL tools were used to fix the number of rotatable bonds of the ligand molecules and finally to prepare the corresponding ligand pdbqt files. The targeted docking of the Cipa bioactive compounds at the active site pocket of the 3CL-pro receptor was carried out using grid box size of: 50 Å × 60 Å × 56 Å with corresponding X center, Y center and Z center values of -13.399, 15.281 and 68.785 respectively. The docking was caried out with the grid spacing of 0.374 Å. Amongst the nine docking poses obtained after each run of autodock vina, the best pose was selected for each tested ligands based on their calculated free energy of binding values (∆G_binding_).

The docking of the four aforementioned Cipa bioactive compounds at the other two SARS-CoV2 principal therapeutic targets (Replicating RNA polymerase and Spike RBD) were also carried out similarly. Especially the targeted docking with RBD of SARS-CoV2 spike protein was initiated after identification of the ACE2/RBD interface, obtained through PDBePISA analysis of the crystal structure, 6M0J. Accordingly, the targeted docking was carried out using grid box size of 32 Å × 90 Å × 58 Å with corresponding X center, Y center and Z center values of 6.743, -18.516 and 68.846 respectively. The docking was caried out with the grid spacing of 0.374 Å and the best pose of each tested ligand was selected based on the calculated free energy of binding (∆G_binding_).

Prior to the docking simulation of the principal Cipa bioactive compounds with the RBD of SARS-CoV2 delta variant, the receptor spike protein was modelled by homology modelling (Fig. S4). For the homology modelling of delta (B.1.617.2) variant of SARS-CoV2 spike protein, the sequence information was retrieved from Uniprot [19]. Spike mutations found in Delta Variant include T19R, (V70F*), T95I, G142D, E156-, F157-, R158G, (A222V*), (W258L*), (K417N*), L452R, T478K, D614G, P681R, D950N [20]. The mutant sequence was generated by introducing these amino-acid substitutions in the native spike sequence, which was later used for generating models using Modeller [21]. Once again, Autodock Vina was used to carry out the docking simulation with the grid box size of of 92 Å × 86 Å × 82 Å and with corresponding X center, Y center and Z center values of 147.356, 141.304 and 59.176 respectively.

The receptor-ligand interactions involved in every docked complex were visualized using Pymol [18] and LigPlot + [22].

## Results

### Cissampelos pareira L. shows high positive connectivity with small compounds predicted to inhibit SARS-CoV-2

To assess which among the numerous small compounds predicted to have antiviral potential against SARS-CoV2 in various studies might have similar signatures among the same cell lines as *Cipa*, we queried the connectivity map. We observed 7 small compounds to possess high signature similarity with *Cipa*. These include emetine (99.61), anisomycin (99.58), cycloheximide (99.86), homoharringtonine (99.51), apcidin (86.32), ruxolitinib (91.54), and sirolimus (95.45). These small compounds were predicted using different methods in four independent studies (Table 1).

**Table 1:** Connectivity scores of small compounds with repurposing potential against SARS-CoV-2 as predicted by various studies

Compounds	Score	Reference
**Emetine**	**99.61**	(Dyall et al., 2014) [23]
Chloroquine	-45.51	
mefloquine	-3.93	
Amodiaquine	-13.11	
Gemcitabine	1.59	
Tamoxifen	-23.6	
Toremifene	-17.23	
Terconazole	-14.17	
**Anisomycin**	**99.58**	
**cycloheximide**	**99.86**	
**Homoharringtonine**	**99.51**	
Fluspirilene	-35.73	
Thiothixene	-1.66	
Fluphenazine	17.51	
Chlorpromazine	-77.85	
Triflupromazine	-0.35	
Clomipramine	-11.65	
Imatinib	-1.34	
Dasatinib	-75.92	
Vidarabine	-2.82	(Micholas and Jeremy C., 2020) [24]
eriodictyol	-13.82	
phenformin	1.23	
**Apicidin**	**86.32**	(Gordon et al., 2020) [4]
Haloperidol	-0.74	
Entacapone	6.41	
Metformin	4.37	
H-89	-82.31	
Ribavirin	7.17	
Midostaurin	48.43	
**Ruxolitinib**	**91.54**	
Daunorubicin	-73.76	
Captopril	13.92	
Chloramphenicol	-21.3	
Linezolid	17.29	
Irbesartan	1.09	(Y. Zhou et al., 2020) [25]
Equilin	22.26	
Mesalazine	7.09	
Mercaptopurine	6.02	
Paroxetine	-1.84	
**Sirolimus**	**95.45**	
Carvedilol	-13.58	
Dactinomycin	-39.25	
Eplerenone	10.74	
Oxymetholone	-2.34	

### Cipa whole extract and single molecule constituents can inhibit SARS-COV-2 in vitro

**Fig. 1:**
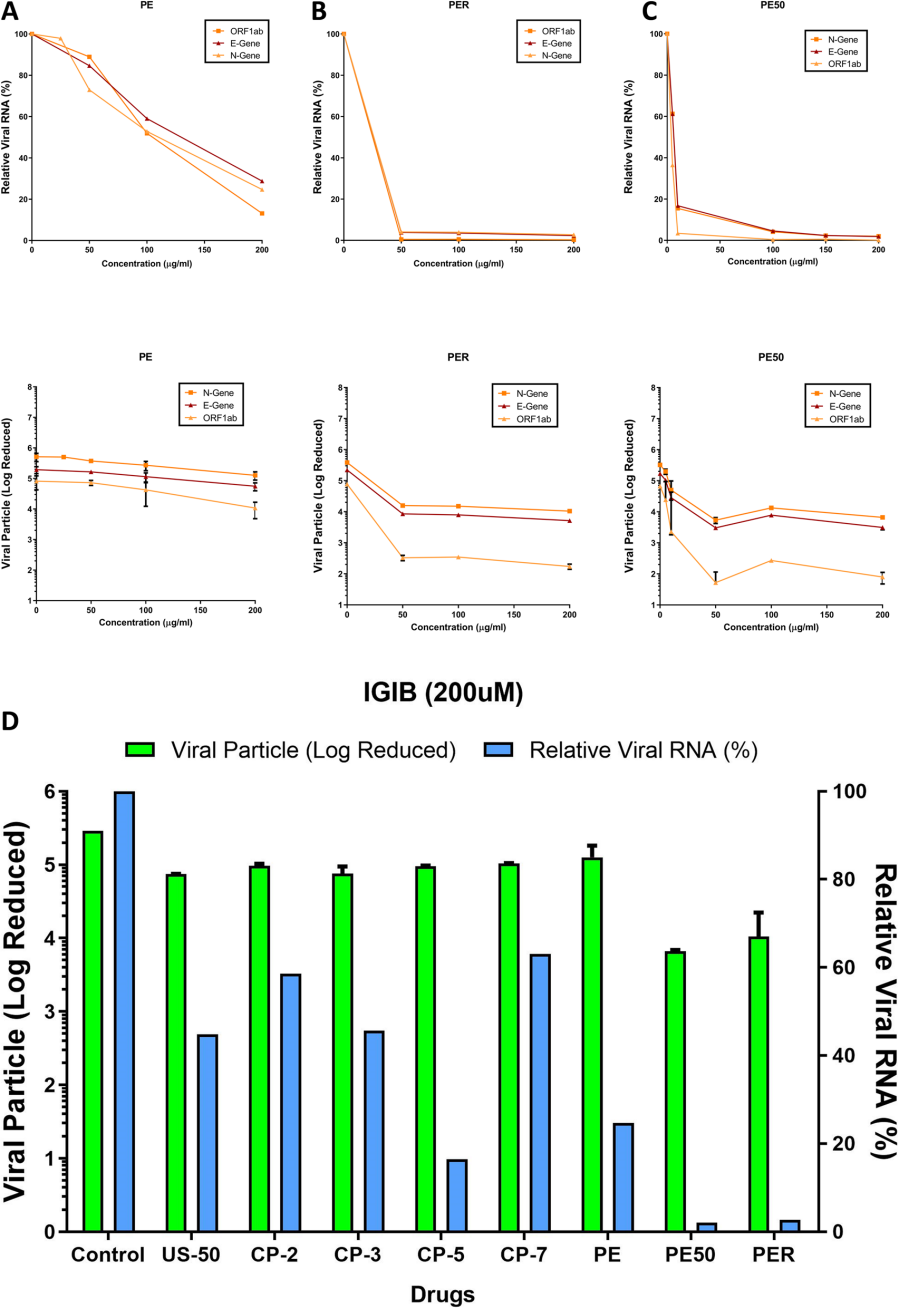
Inhibition of SARS-CoV-2 in vitro by Cipa whole extract and its constituents: Relative viral RNA % and Log reduction in viral particles in vero cells upon treatment at 50, 100, 150 and 200 μg of **A**) whole plant aqueous extract (PE), **B**) root extract (PER) and **C**) hydro-alcoholic extracts (PE50) of Cipa. **D** Sars-cov-2 viral titers inhibition by Cipa constituents CP-2 Salutaridine, CP-3 Cissamine, CP-5 pareirarine, CP-7 Magnoflorine, PE aqueous extract, PE50 50% hydroalcoholic extract and PER root extract. The error bars have been represented in the figures with (*p* < 0.05) and the experiments were done in triplicates

Since metanalysis highlighted an inhibitory potential of *Cipa* against SARS-COV-2, we tested the effect of whole plant and root extracts of *Cipa* in Vero cell culture assays infected with SARS-CoV-2. The relative viral RNA (%) was calculated by considering the values averaged from N (Nucleoprotein), ORF1ab (19 non-structural proteins, NSP1-16), and E (Envelope) viral genes. The whole plant aqueous extract showed a definite antiviral activity, evidenced by decreased relative viral RNA content with a reduction by 57% at 100 µg/ml where the viral particle number reduced from 10^5.9^ to 10^5.6^ (Fig. 1A-C).

**Fig. 2:**
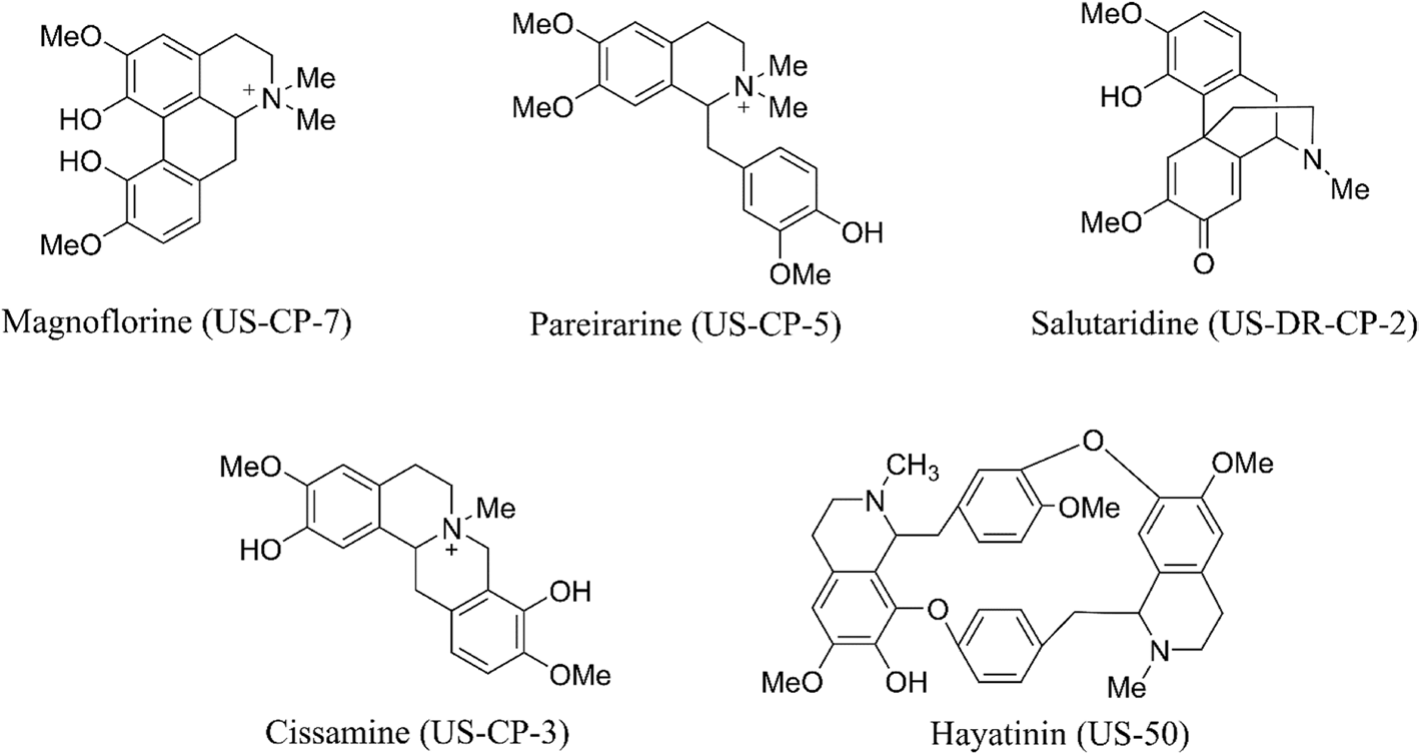
Molecular structures of the major components of the compounds isolated from Cipa and used to determine anti-SARS-CoV-2 activity

Next, we wanted to see whether the *Cipa* pure constituents can inhibit SARS-COV-2. The total alkaloid content in the extract was 46.4 mg/g with cissamine (18.6 mg/g) being the major one, followed by magnoflorine (12.9 mg/g). The pure molecules namely hayatinin (US-50), salutaridine (US-DR-CP-2), cissamine (US-CP-3), pareirarine (US-CP-5), magnoflorine (US-CP-7), aqueous whole plant extract (PE), 50% hydroalcoholic whole plant extract (PE50) and 50% hydroalcoholic root extract (PER) were tested against SARS-CoV2 at 200 µM concentration showed relative viral RNA (%) to 44, 58, 45, 16, 63, 24, 2 and 2 respectively in comparison with the virus control (Fig. 1D). The structure of these constituent compounds is given in Fig. 2.

### Cipa bioactive compounds bind pivotal SARS-CoV2 therapeutic targets

**Fig. 3:**
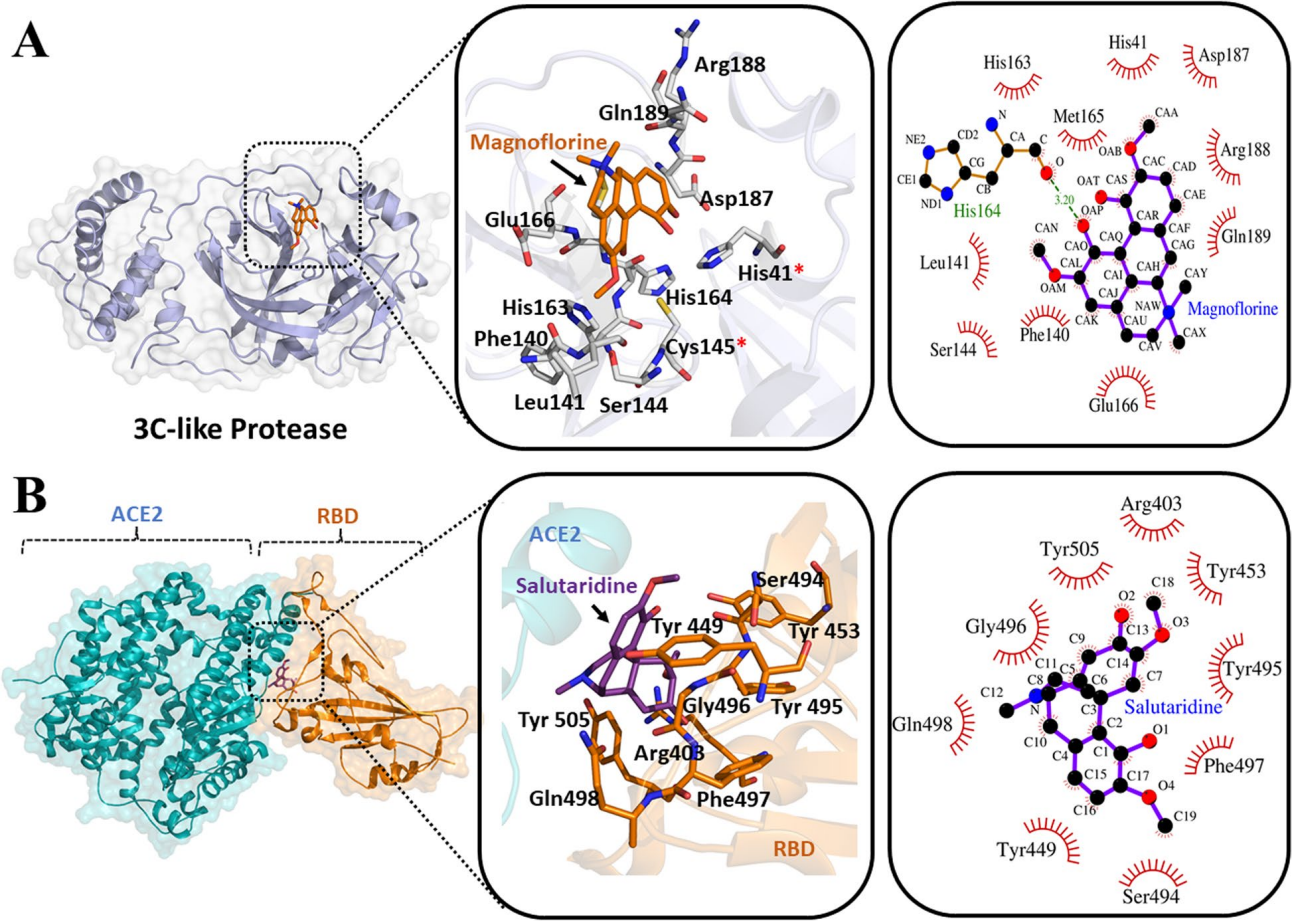
*Cipa* principal bioactive compounds bind crucial SARS-CoV2 therapeutic targets. **A** represents molecular docking of Magnoflorine at the active site pocket of SARS-CoV2 3C-like protease. **B** represents molecular docking of Salutaridine with ACE2 interacting amino acid residues of Receptor Binding Domain (RBD) from SARS-Cov2 spike protein. The middle and the right panels of each figure represent the zoomed in view of the molecular interaction patterns of the docked ligands in three-dimensional and two-dimensional spaces respectively. In the two-dimensional interaction plots, the green dotted lines indicate hydrogen bond formation, whereas the hydrophobic interactions are represented by the spiked arcs

In order to establish the molecular mechanism of action of principal Cipa bioactive compounds which manifested SARS-CoV2 inhibition in vitro (Magnoflorine, Pareiramine, Salutaridine and Cissamine), we tested their ability to bind principal drug target proteins of SARS-CoV2 in silico (3C like protease, replicating RNA polymerase and Receptor Binding Domain of viral spike protein). Amongst all the tested compounds, Magnoflorine and Salutaridine exhibited the most potent and consistent strong in silico binding profiles with SARS-CoV2 therapeutic targets (Fig. 3). Magnoflorine was found to bind SARS-CoV2 3C-like protease (3CL-pro) with excellent profile of ∆G_binding_ (-7.3 kcal/mol) comparable to GC373 (-7.3 kcal/mol), one of the well-established synthetic 3CL-pro inhibitor [15] tested as the positive control under the same docking simulation conditions. Moreover, Magnoflorine and GC373 share the common binding sites at the evolutionary conserved active site catalytic groove of 3CL-pro (Fig. 3A and Fig. S2) in close proximity of the Cys145-His41 catalytic dyad. Both of these tested compounds bind the active site amino acid residues of 3CL -pro through an extensive array of hydrogen bonding and hydrophobic interaction networks.

Intriguingly, all the tested Cipa bioactive compounds were found to dock with the Receptor Binding Domain (RBD) of SARS-CoV2 spike protein (Fig. S3), another crucial target for anti-SARS-CoV2 therapeutic intervention. The free energy of binding (∆G_binding_) ranges from -7.1 kcal/mol to -5.6 kcal/mol from best to worst binding ligands. Amongst the all tested compounds, Salutaridine was found to exhibit the most potent and consistent strong binding profile (∆G_binding_ -7.1 kcal/mol) (Fig. 3B). Importantly, the binding of Cipa ligands at the RBD engages the same set of amino acid residues which are responsible for the SARS-CoV2 spike interaction with receptor ACE2 (PDB ID: 6M0J). Moreover, Salutaridine binding at the RBD is found to be structurally reminiscent with comparable free energy of binding of H69D1(∆G_binding_ -6.8 kcal/mol), a potent spike protein targeting SARS-CoV2 entry blocker (used as the positive control) [26]. The free energy of binding of all tested Cipa bioactive compounds at the RBD-ACE2 interface stabilizing residues are also found to be energetically favourable compared to the free energy of dissociation of RBD-ACE2 complex [∆G^diss^ -2.00 kcal/mol; calculated by PDBePISA [27]]. Therefore, all the four Cipa bioactive compounds tested herein can potentially function as SARS-CoV2 entry blocker targeting the RBD of the spike protein.

Unlike the active site catalytic residues of 3CL -pro, the ACE2 interacting residues of RBD are highly variable amongst different SARS-CoV2 pathovariants. In this context, in silico docking of the same set of Cipa bioactive compounds was carried out against the RBD of SARS-CoV2 delta variant. Importantly, in case of the delta variant, the tested Cipa bioactive compounds were found to strongly bind at the distal drug binding hotspot of RBD [28–30] (Fig. S4).

### Cipa transcriptome oppositely regulates several biological pathways compared to COVID-19 infected patient BALF transcriptome

**Fig. 4:**
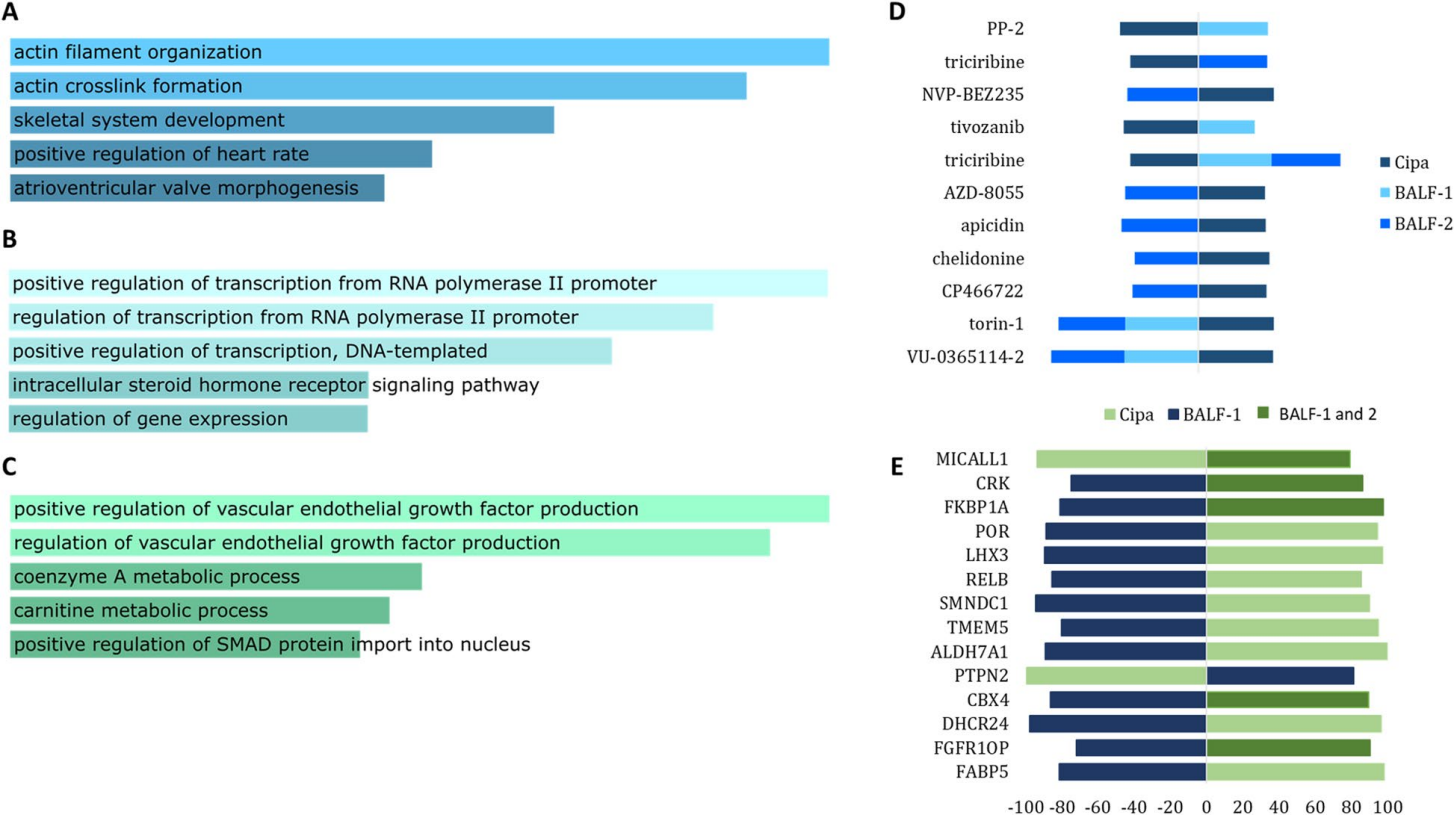
Comparative analysis of Cipa with BALF-1 and BALF-2 transcriptome: Functional enrichment of genes common between **A**) Cipa and BALF-1, **B**) Cipa and BALF-2 and **C**) Cipa, BALF-1 and BALF-2. **D** Small compound signatures common between all three, the space on the left of the vertical axis indicates negative signatures while the one on the right indicates positive signatures. **E** Genetic signatures common between all three, the space on the left of the vertical axis indicates negative signatures while the one on the right indicates positive signatures

We looked for the differentially expressed genes which were common between *Cipa* and BALF samples from two studies [31, 32], hereby referred to as BALF-1 and BALF-2 respectively. We observed that 39 genes were common between *Cipa* and BALF-1 and BALF-2, out of which 29 showed an opposite expression (Table S1). Individually, 134 genes were common between *Cipa* and BALF-1, and 174 genes common between *Cipa* and BALF-2. Upon functional enrichment analysis we observed that the genes enriched for regulation of vascular endothelial growth were upregulated in both BALF-1 and 2 datasets while being downregulated by *Cipa*. Similarly, while regulation of gene expression was upregulated by *Cipa* it was downregulated in BALF-1 and BALF-2 data sets (Fig. 4A-C).

The connectivity map analysis of the gene signatures of BALF-1 and BALF-2 comparison with *Cipa* results revealed a number of small compounds that had high positive scores with *Cipa* and negative scores with either BALF transcriptomes. We observed several small compounds having opposite signature similarities between *Cipa* and BALF. Triciribine, torin-1 and VU-0365114–2 were three compounds with negative scores for *Cipa* and positive scores for both BALF 1 and 2 (Fig. 4D). Also, knockdown signatures of a number of genes were found to exhibit opposing scores between Cipa and BALF transcriptomes. Some of these include, MICALL1, CRK, FKBP1A, CBX4, and FGR10P (Fig. 4E).

## Discussion

SARS-COV-2 has shown a very diverse set of clinical presentations in various populations and among genders within the same population. It has been shown that estrogen can regulate the expression of ACE-2 receptors [5]. Although a few known herbal medicines such as extracts of *Ganoderma lucidum*, *Perilla frutescens*, and *Mentha haplocalyx* have been shown to be effective against SARS-CoV-2 infection [33], only *Ganoderma lucidum* has shown effect on estrogen modulation [34]. Since *Cipa* appears to have *ESR1* modulatory effects [10], and has been shown to have antiviral potential [8], we hypothesized it may have inhibitory effect on the novel coronavirus. CMAP analysis of *Cipa* transcriptome signatures highlights several small compounds having been predicted to have inhibitory activity against SARS-CoV-2. Among these, emetine, homoharringtonine, and cycloheximide are known translation inhibitors. These have also been shown to inhibit Zika and Ebola [35], SARS and MERS [23], and Newcastle disease virus [36]. Apcidin, an HDAC inhibitor has been predicted to inhibit SARS-CoV-2 in a recent study [37].

In vitro experiments for viral inhibition of SARS-COV-2, reveal that all whole plant extracts of *Cipa* (aqueous and alcoholic) could inhibit the virus at least up to 60%. Hydroalcoholic whole plant extract showed an inhibition of 98%. The single molecule constituents of Cipa could also inhibit the viral particles, with pareirarine showing the highest inhibition of 80%. This showed that *Cipa* does have the potential to inhibit SARS-CoV-2 virus in vitro. Interestingly, the highest inhibition is shown by the whole plant hydroalcoholic extract which comprises of various small compound constituents. This suggests a synergistic effect of the constituents towards viral inhibition. Interestingly, among the tested constituent compounds, pareiraine was reported for the first time from *Cissampelos pareira* in a study carried for antiplasmodial activity [13]. Cissamine belongs to the protoberberine class of isoquinoline alkaloids and antiviral activity of cissamine was reported here for the first time. But berberine being the most prominent compound of protoberberine class exhibited antiviral activity against HCV, HPV, HIV-1 [38] and H1N1 influenza A virus [38, 39]. Magnoflorine belongs to the aporphine class of isoquinoline alkaloids and such types of alkaloids are reported to have antiviral activity against Herpes simplex virus [40] and human poliovirus [41]. However, there are no individual reports regarding the antiviral activity of magnoflorine. In one study, methanolic leaves extract of *Magnolia grandiflora* containing magnoflorine was observed to have high antiviral activity against Herpes simplex virus (HSV-1) and possessed moderate antiviral activity against poliovirus type-1 (PV1) [42].

In silico ligand binding assays clearly indicates the molecular therapeutic mechanism of Cipa bioactive compounds against the tested SARS-CoV2 drug targets. The in silico results presented herein corroborates with in vitro SARS-CoV2 inhibition properties of the tested compounds. Amongst the all tested Cipa compounds against three major SARS-CoV2 therapeutic targets, the 3CL -pro binding inhibitor, holds immense promise in context of its highly conserved binding site in contrast to the rapidly evolving ACE2 binding landscape of the RBD in different viral pathovariants. Furthermore, the observed synergistic antiviral property of Cipa hydroalcholic extracts in vitro, may result from the unique ability of the constituent bioactive molecules to simultaneously target several SARS-CoV2 therapeutic targets as observed in the in silico docking experiments presented here. Importantly, other than the three major viral therapeutic targets tested here, multiple crucial host derived proteins/protein complexes also play trivial role in SARS-CoV2 disease pathogenesis. Structural investigation of bioactive ligand binding to host derived therapeutic targets would further open up new arenas of future anti-SARS-CoV2 therapeutic inventions.

We also found that the signatures of the transcriptomic changes in *Cipa* treated MCF7 cells and BALF from patients’ lungs, have interesting overlaps. Among the connected small compounds triciribine, torin-1 and VU-0365114–2, triciribine has been shown to inhibit Human Immunodeficiency virus sera types 1 and 2 [43]. Another study has shown that VU-0365114–2, which is a muscarinic acetylcholine receptor M5 inhibitor, has repurposing potential against SARS-CoV-2 [44]. While mTOR inhibitor torin-1 may modulate immune activity and enhance antiviral response, even against SARS-COV-2 [45].

There are a number of factors which modulate the viral-host interactions which can be explored for better understanding the mechanism of action of promising antivirals such as interactions with the membrane components such as lipid rafts and ACE2, and other key non-structural proteins of the virus [6, 46]. Interestingly, GPER, the G-protein coupled estrogen receptor is often found associated with lipid rafts and have been reported to modulate estrogenic effects in the immune cells [47, 48]. It is worthwhile to note that in our previous study [10], we found that *Cipa* can down regulate this receptor as well. Since estrogen has been reported to effect covid related disease progression and fatality [5, 49, 50], it will be prudent to study the interaction of GPER and associated lipids in the plasma membrane with viral proteins and explore their potential as drug targets.

## Conclusion

In summary, we report here a framework applicable for repurposing of herbal formulations that are used in Ayurveda and other medicinal system using an integrated multi-pronged approach using transcriptome-based connectivity mapping, in vitro validation and conjoint analysis with disease signatures. We demonstrate the potential repurposing of *Cissampelos pareira* L for SARS-CoV-2 using this approach.

## Supplementary Information

**Additional file 1:** **Supplementary methods. Fig. S1.** Flow chart of preparation of extracts, and isolation of pure molecules from Cissampelos pareira. **Fig S2.** Magnoflorine is a plausible SARS-CoV2 3C-like protease inhibitor. The left panel exhibits Magnoflorine binding at evolutionary conserved active site catalytic groove of SARS-CoV2 3C-like protease. The colour coded surface map represents site specific amino acid evolution rate of SARS-CoV2 3C-like protease. The middle panel and the right panel represent the mutually exclusive binding site of Magnoflorine and GC373, a well-established synthetic inhibitor of SARS-CoV2 3C-like protease [Vuong W, Khan MB, Fischer C, Arutyunova E, Lamer T, Shields J, et al. Feline coronavirus drug inhibits the main protease of SARS-CoV-2 and blocks virus replication. Nat Commun. 2020;11:4282]; encircled amino acid residues in the right panel are the common interaction partners for Magnoflorine and GC373. **Fig S3.** Cipa principle bioactive compounds (Salutaridine, Pareiramine, Magnoflorine, Cissamine) are plausible spike protein targeting cellular entry inhibitor of SARS-CoV2. Docked bioactive compounds and H69D1, an established synthetic SARS-CoV2 entry blocker [Wang L, Wu Y, Yao S, Ge H, Zhu Y, Chen K, et al. Discovery of potential small molecular SARS-CoV-2 entry blockers targeting the spike protein. Acta Pharmacol Sin. 2021;:1–9] share the same amino acid binding partners from the Receptor Binding Domain (RBD) of SARS-CoV2 spike protein. Left and middle panels show the interaction of tested Cipa bioactive compounds and H69D1 at the ACE2/RBD interface formed during SARS-CoV2 cellular entry (PDB ID: 6M0J). In the right panel, encircled amino acid residues in the two-dimensional interaction plot are the common interaction partners for the tested Cipa bioactive compounds and H69D1. **Fig S4.** Cipa bioactive compounds bind spike protein of SARS-CoV2 delta variant at a distal drug binding hotspot. Table S1: 39 genes common between Cipa, BALF-1 and BALF-2.

## Data Availability

The raw data analysed during the current study are available in publicly available at respective repositories and data centers as follows: Transcriptomic data of Cissampelos pareira: GEO public repository: GSE156445 [https://www.ncbi.nlm.nih.gov/geo/query/acc.cgi?acc=GSE156445]. Transcriptomic data of SARS-COV-2 BALF: GEO public repository: GSE152075 [https://www.ncbi.nlm.nih.gov/geo/query/acc.cgi?acc=GSE152075] and Genome Warehouse in National Genomics Data Center: PRJCA002273 [https://ngdc.cncb.ac.cn/gsa-human/browse/HRA000143].

## Abbreviations

CMAP: Connectivity Map
BALF: Bronchoalveolar Lavage fluid
ESR1: Estrogen Receptor Alpha
ACE2: Angiotensin-converting enzyme 2
SARS: Severe Acute Respiratory Syndrome
MERS: Middle East respiratory syndrome
HDAC: Histone deacetylase
