# Supplementary material for: Anti-SARS-CoV-2 potential of Cissampelos pareira L. identified by connectivity map-based analysis and in vitro studies
Source: BMC Complement Med Ther. 2022 Apr 22;22:114. doi: 10.1186/s12906-022-03584-3 (PMC9028906; doi:10.1186/s12906-022-03584-3)
Supplement: Supplementary file 1 — Additional file 1: Supplementary methods. Fig. S1. Flow chart of preparation of extracts, and isolation of pure molecules from Cissampelos pareira. Fig S2. Magnoflorine is a plausible SARS-CoV2 3C-like protease inhibitor. The left panel exhibits Magnoflorine binding at evolutionary conserved active site catalytic groove of SARS-CoV2 3C-like protease. The colour coded surface map represents site specific amino acid evolution rate of SARS-CoV2 3C-like protease. The middle panel and the right panel represent the mutually exclusive binding site of Magnoflorine and GC373, a well-established synthetic inhibitor of SARS-CoV2 3C-like protease [Vuong W, Khan MB, Fischer C, Arutyunova E, Lamer T, Shields J, et al. Feline coronavirus drug inhibits the main protease of SARS-CoV-2 and blocks virus replication. Nat Commun. 2020;11:4282]; encircled amino acid residues in the right panel are the common interaction partners for Magnoflorine and GC373. Fig S3. Cipa principle bioactive compounds (Salutaridine, Pareiramine, Magnoflorine, Cissamine) are plausible spike protein targeting cellular entry inhibitor of SARS-CoV2. Docked bioactive compounds and H69D1, an established synthetic SARS-CoV2 entry blocker [Wang L, Wu Y, Yao S, Ge H, Zhu Y, Chen K, et al. Discovery of potential small molecular SARS-CoV-2 entry blockers targeting the spike protein. Acta Pharmacol Sin. 2021;:1–9] share the same amino acid binding partners from the Receptor Binding Domain (RBD) of SARS-CoV2 spike protein. Left and middle panels show the interaction of tested Cipa bioactive compounds and H69D1 at the ACE2/RBD interface formed during SARS-CoV2 cellular entry (PDB ID: 6M0J). In the right panel, encircled amino acid residues in the two-dimensional interaction plot are the common interaction partners for the tested Cipa bioactive compounds and H69D1. Fig S4. Cipa bioactive compounds bind spike protein of SARS-CoV2 delta variant at a distal drug binding hotspot. Table S1: 39 genes common between Cipa, BALF-1 [file 12906_2022_3584_MOESM1_ESM.docx]

**Supplementary Information for:**

**Anti-SARS-CoV-2 potential of *Cissampelos pareira* L. identified by Connectivity map-based analysis and in vitro studies**

Madiha Haider^a,b^ Vivek Anand^a,b^ M. Ghalib Enayathullah^c^ Yash Parekh^c^, Sushma Ram^c^ Surekha Kumari^d,b^ Anmol^d,b^ Gayatri Panda^e^ Manjari Shukla^g^ Dhwani Dholakia^a,b^ Arjun Ray^e^ Sudipta Bhattacharyya^g^ Upendra Sharma^d,b^ Kiran Kumar Bokara^c^ Bhavana Prasher^*a,b,f^ Mitali Mukerji ^*a,b,f,g^

^a^Genomics & molecular medicine, CSIR-Institute of Genomics and Integrative Biology, Delhi, India-110007, ^b^Academy of Scientific and Innovative Research, Ghaziabad, Uttar Pradesh, India 201002, ^c^CSIR-Center for Cellular and Molecular Biology, Hyderabad, Telangana, 500007, India, ^d^Chemical Technology Division, CSIR-Institute of Himalayan Bioresource Technology, Palampur, Himachal Pradesh 176 061, ^e^3Indraprastha Institute of Information Technology, Delhi, India ^f^Centre of excellence for Applied developments of Ayurveda prakriti and genomics, CSIR’s Ayurgenomics Unit TRISUTRA, CSIR-IGIB, India, ^g^Department of Bioscience & Bioengineering, Indian Institute of Technology Jodhpur, NH 62, Karwar, Rajasthan 342037

*Corresponding Authors

Genomics & molecular medicine, CSIR-Institute of Genomics and Integrative Biology, Delhi, India-110007

**Email:** [bhavana.p@igib.res.in](mailto:bhavana.p@igib.res.in)

**Email:** mitali@iitj.ac.in

^g^Current address: Department of Bioscience & Bioengineering, Indian Institute of Technology Jodhpur, NH 62, Karwar, Rajasthan 342037

**1. Supplementary methods:**

**1.1 SARS-CoV-2 transcriptome meta-analysis:**

RNA-Seq sample pre-processing:

Created annotated transcripts from human genome (GRCh37) and GTF file (gencode v19) using gffread (v0.12.1) and further indexing the transcripts using Salmon index (v1.3.0).

salmon index --threads $cpus -t $fasta --gencode -i salmon_index 2

Raw reads were mapped and quantified using salmon quant with options --validateMappings (**improve quantification accuracy**), --seqBias (**correct sequence-specific bias**), --useVBOpt (**use Bayesian EM algorithm**), --gcBias (**correct GC bias**).

salmon quant --validateMappings --seqBias --useVBOpt --gcBias --geneMap $gtf --threads $cpus --libType=A --index $salmon_index -1 $reads1.fq -2 $reads2.fq --writeUnmappedNames -o $sample

Salmon gives transcript level abundance for each sample and later converted into gene-level expression using tximport (v1.16.1). Before moving to differential gene expression, Surrogate Variable Analysis (v3.36.0) in an unsupervised setting to correct the batch effect in the samples considering the sequencing machine bias in both groups.

**1.2 Preparation of *Cissampelos pareira* L. extracts and isolation of small compounds:**

**Chemicals and reagents:** For UPLC analysis formic acid was obtained from S. D. Fine Chemicals Ltd. (Mumbai, India) whereas methanol and water (LC grade) were purchased from J. T. Baker (Mallinckrodt Baker Inc., St. Louis, MO, USA).

**Preparation of Extracts and Isolation of Pure Compounds:** The collected plant material was dried using shade drying method. The air dried whole plant (root, stem and leaves) material (230 g) was extracted thrice with ethanol: water (50: 50) by percolation. The percolate was evaporated to dryness in rotary evaporator at temperature 50 °C to obtain 25.0 g (10.8% w/w) crude extract (USCPWP-PE-50). The air dried roots (950 g) were extracted thrice with ethanol: water (80: 20) by percolation at room temperature. The percolate was evaporated in rotary evaporator at temperature 50 °C to yield 98.0 g (10.3 % w/w) crude extract (USCPR-PE-R) (Fig. S1). The whole plant aqueous extract was obtained commercially from a GMP certified manufacturer.

**Quantitative analysis of the samples:** The marker compounds in crude extract (USCPWP-PE-50) were quantified by the UPLC-DAD method reported recently [1]**.**


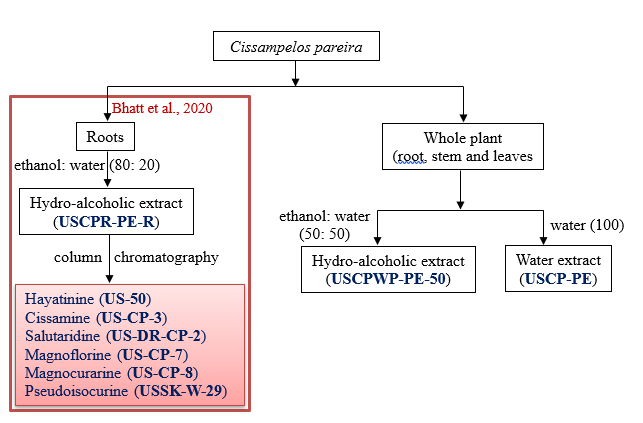


**Fig. S1: Flow chart of preparation of extracts, and isolation of pure molecules from Cissampelos pareira.**

**1.3 Cell culture, viral infection and drug treatment for inhibition of SARS-COV-2 by Cipa:**

The cells were maintained in Dulbecco Minimum Essential Medium (DMEM) (Gibco) containing 10% Fetal Bovine Serum (FBS) (Gibco) at 37°C, 5% CO2. Initially the compounds were dissolved in organic and aqueous solvents based on the requirements, and stocks were made. The concentrations (200, 100, 50, 10, 5 (µg/mL), were made using the DMEM media. Briefly, the cells were pre-incubated with the compounds (PE-50 & R) for 2 hours, for each concentration in triplicate. Later, the virus inoculum (at a 0.1 MOI) was added to the cells for 3 hours in presence of the respective dilutions of compounds made in Basal medium only. Post-infection, viral inoculum was replaced with fresh media containing 10% FBS and the experiment is continued in the presence of different dilutions of compounds for 72 hours. After 72 hours, cell supernatant was collected and spun for 10 min at 6,000 g to remove debris and the supernatant was transferred to fresh collection tubes for further analysis.

Isolation of Viral RNA:

RNA was isolated from 200 μL of the supernatants using the The viral supernatants from the test groups were added into the deep well plate (KingFisherTM Thermo Scientific) along with a lysis buffer containing the following components - MagMAXTM Viral/Pathogen Binding Solution (260μL); MVP-II Binding Beads (10 μL); MagMAXTM Viral /Pathogen Proteinase-K of (5 μL) respectively. RNA extraction was performed using KingFisher Flex (version 1.01, Thermo Scientific) by following manufacturer's instructions. The eluted RNA was immediately stored in -80⁰C until further use.

**1.4 TaqMan Real-time RT-PCR assay for Detection of SARS-CoV-2:**

The kit detects Envelope gene (E; ROX labelled), Nucleocapsid gene (N- JOE labelled) and open reading frame 1ab (ORF1ab, FAM labelled) specific to SARS-CoV2 for detection and amplification of the cDNA. Briefly, RT-qPCR assays were performed on a Quant studio Q5 (Thermo fisher). A 10 μL of Negative Control, 10 μL of Positive Control (positive and negative controls were provided by kit), and 10 μL of extracted RNA from samples were added in different PCR reaction tubes. The contents were centrifuged at low speed. The cycling conditions are: Step 1: 50°C for 15 minutes, 1 cycle; Step 2: 95°C for 3 minutes, 1 cycle; Step 3: 95°C for 5 seconds to 60°C for 40 seconds, 5 cycles; Step 4: 95°C for 5 seconds to 60°C for 40 seconds, 40 cycles. The signals of FAM, JOE, ROX and CY5 (internal reference) fluorescence channels were collected at 60°C. SARS-CoV-2 cDNA (Ct~28) was used as a positive control. The log viral particles and a semi–log graph was plotted using Graph Pad Prism 5 software (ver 5.03).

**2. Supplementary Figures:**


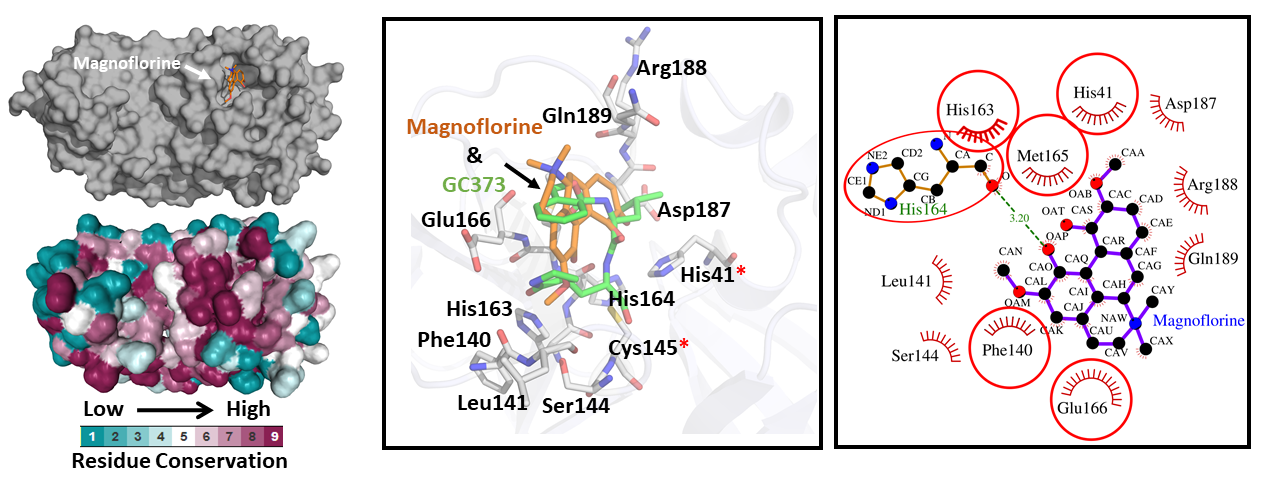


**Fig S2:** Magnoflorine is a plausible SARS-CoV2 3C-like protease inhibitor. The left panel exhibits Magnoflorine binding at evolutionary conserved active site catalytic groove of SARS-CoV2 3C-like protease. The colour coded surface map represents site specific amino acid evolution rate of SARS-CoV2 3C-like protease. The middle panel and the right panel represent the mutually exclusive binding site of Magnoflorine and GC373, a well-established synthetic inhibitor of SARS-CoV2 3C-like protease [2]; encircled amino acid residues in the right panel are the common interaction partners for Magnoflorine and GC373.


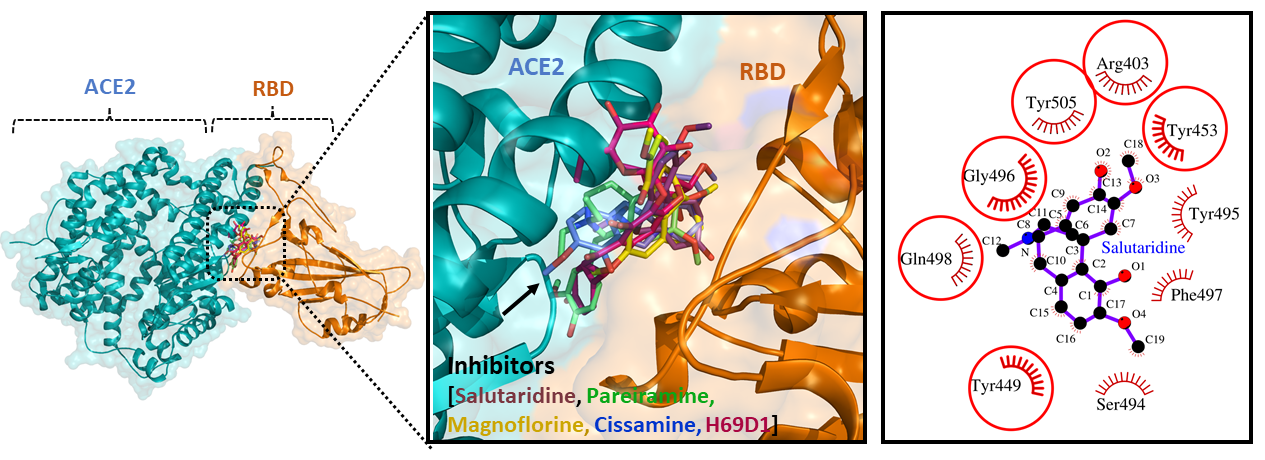


**Fig S3:** Cipa principle bioactive compounds (Salutaridine, Pareiramine, Magnoflorine, Cissamine) are plausible spike protein targeting cellular entry inhibitor of SARS-CoV2. Docked bioactive compounds and H69D1, an established synthetic SARS-CoV2 entry blocker [3] share the same amino acid binding partners from the Receptor Binding Domain (RBD) of SARS-CoV2 spike protein. Left and middle panels show the interaction of tested Cipa bioactive compounds and H69D1 at the ACE2/RBD interface formed during SARS-CoV2 cellular entry (PDB ID: 6M0J). In the right panel, encircled amino acid residues in the two-dimensional interaction plot are the common interaction partners for the tested Cipa bioactive compounds and H69D1.


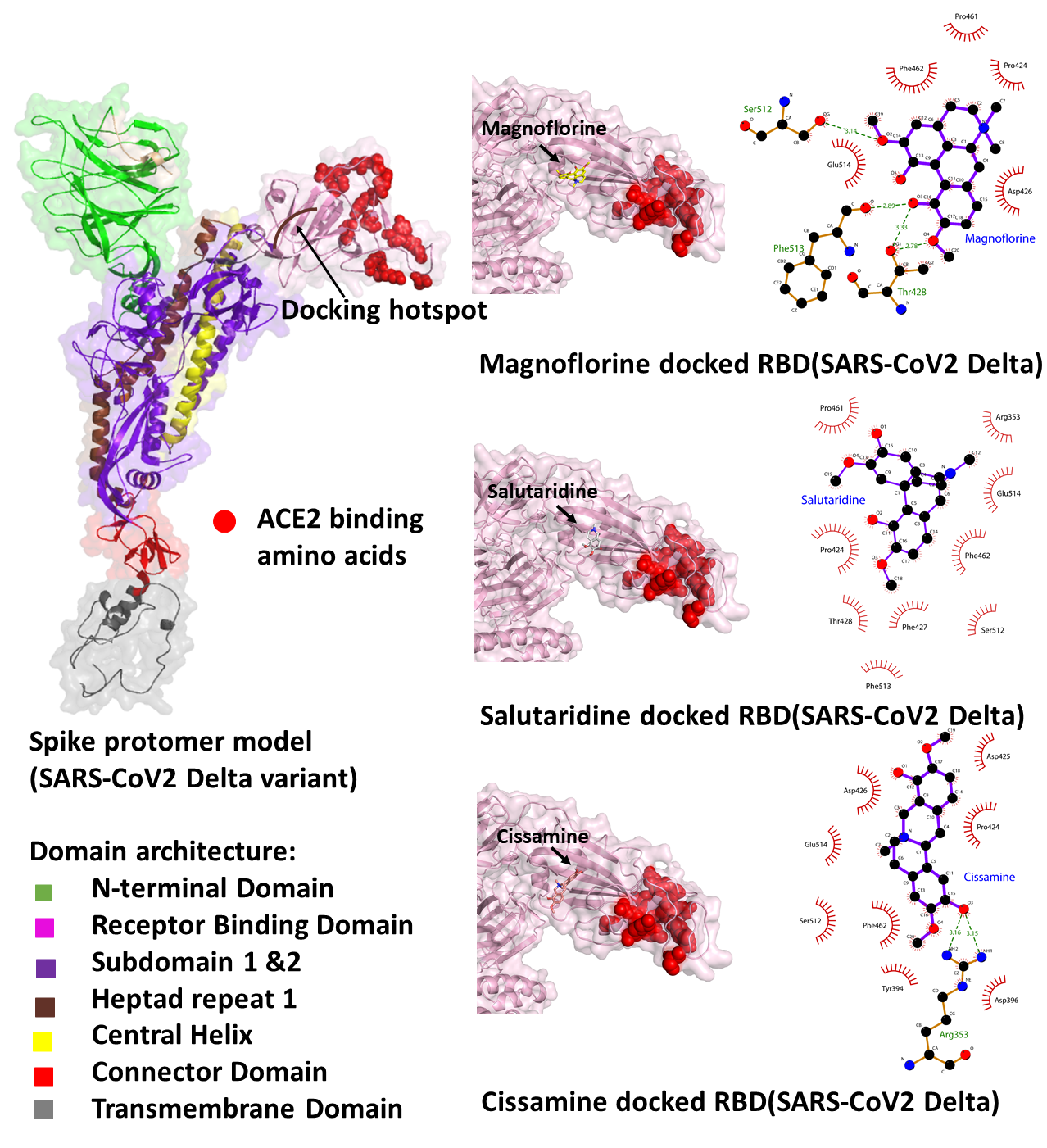


**Fig S4:** Cipa bioactive compounds bind spike protein of SARS-CoV2 delta variant at a distal drug binding hotspot.

**3. Supplementary table:**

**Table S1: 39 genes common between Cipa, BALF-1 and BALF-2**

|  | Cipa | BALF-1 | BALF-2 |
| --- | --- | --- | --- |
| AGR3 | -1.38746 | -1.18033 | -4.78508 |
| MSMB | -1.33061 | 1.070106 | -5.04154 |
| ACLY | -1.30041 | 0.295665 | 3.559965 |
| PBX1 | -1.24767 | 1.487727 | 3.468367 |
| PDIA4 | -1.24474 | 0.117456 | 6.354455 |
| CROT | -1.20234 | -0.83981 | 3.680022 |
| C1orf21 | -1.17113 | -1.92896 | 2.874781 |
| SCAI | -1.1355 | -0.72477 | 4.309578 |
| BCL9 | -1.1204 | 0.064614 | 4.146401 |
| PORCN | -1.08959 | -0.59788 | 3.219026 |
| EVL | -1.08953 | -1.47847 | 7.791948 |
| TTLL5 | -1.0657 | -0.10307 | 2.754773 |
| ETNK2 | -1.04426 | -2.07727 | 5.625841 |
| TGFB1 | -1.04116 | -0.93531 | 2.21809 |
| ANTXR1 | -1.03224 | -1.01169 | 3.570147 |
| TET1 | -1.032 | -0.90988 | 5.321577 |
| NUBPL | -1.01577 | -0.12047 | 4.078702 |
| SBK1 | -1.01475 | -2.39136 | 2.946711 |
| RBPMS | -1.00924 | -0.88967 | 2.967272 |
| KIAA1217 | 1.012205 | -0.73144 | -2.07887 |
| CBR3-AS1 | 1.030189 | -0.18835 | 3.847575 |
| TBC1D12 | 1.037079 | 1.18878 | 4.152344 |
| RANBP6 | 1.059566 | -0.34571 | 4.111845 |
| STOM | 1.080323 | 0.232034 | 3.092429 |
| SLC22A5 | 1.090643 | -0.38776 | 5.721312 |
| CNKSR3 | 1.116294 | 0.0481 | 4.422747 |
| PLD1 | 1.122033 | 1.093094 | 2.99798 |
| ZNF461 | 1.189381 | -0.68859 | 4.836408 |
| LMO7 | 1.22905 | -1.23364 | -2.8111 |
| CHD2 | 1.277242 | -0.59575 | 2.136096 |
| EIF2AK3 | 1.341297 | 0.10631 | 3.506099 |
| MYO9A | 1.616131 | -0.78824 | 4.192263 |
| PSPC1 | 1.624655 | -0.5562 | 4.292772 |
| NEDD4 | 1.711919 | -0.95851 | 2.477732 |
| ZNF462 | 1.867743 | -3.00318 | 6.111656 |
| MYO1B | 2.048216 | 0.553853 | -2.9671 |
| TAGLN | 2.238096 | -0.80487 | 6.711373 |
| CYP1B1 | 2.547549 | 1.94272 | -4.75062 |
| RND3 | 3.153523 | -1.67392 | -3.60436 |

**References:**

1. Bhatt V, Kumari S, Upadhyay P, Agrawal P, Anmol, Sahal D, et al. Chemical profiling and quantification of potential active constituents responsible for the antiplasmodial activity of Cissampelos pareira. J Ethnopharmacol. 2020;262:113185.

2. Vuong W, Khan MB, Fischer C, Arutyunova E, Lamer T, Shields J, et al. Feline coronavirus drug inhibits the main protease of SARS-CoV-2 and blocks virus replication. Nat Commun. 2020;11:4282.

3. Wang L, Wu Y, Yao S, Ge H, Zhu Y, Chen K, et al. Discovery of potential small molecular SARS-CoV-2 entry blockers targeting the spike protein. Acta Pharmacol Sin. 2021;:1–9.
